# Methyl 4-(3-chloro­prop­oxy)benzoate

**DOI:** 10.1107/S1600536811011093

**Published:** 2011-04-07

**Authors:** Ya-Bin Shi, Ke-Ke Liu, Song Xia, Fei-Fei He, Hai-Bo Wang

**Affiliations:** a College of Food Science and Light Industry, Nanjing University of Technology, Xinmofan Road No. 5 Nanjing, Nanjing 210009, People’s Republic of China; b College of Science, Nanjing University of Technology, Xinmofan Road No. 5 Nanjing, Nanjing 210009, People’s Republic of China

## Abstract

In the crystal structure of the title compound, C_11_H_13_ClO_3_, inter­molecular C—H⋯O hydrogen bonds link the mol­ecules into zigzag chains along the *c* axis.

## Related literature

The title compound is an inter­mediate in the synthesis of 4-(3-(dibutyl­amino)­prop­oxy)benzoyl chloride, which in turn is a useful pharmaceutical inter­mediate that can be used to prepare dronedarone [systematic name *N*-(2-butyl-3-(*p*-(3-(dibutyl­amino)­prop­oxy)benzo­yl)-5-benzofuran­yl)methane­sulfonamide]. For background to the biological activity of dronedarone and the preparation of the title compound, see: Jaseer *et al.* (2010 ▶). For bond-length data, see: Allen *et al.* (1987 ▶).
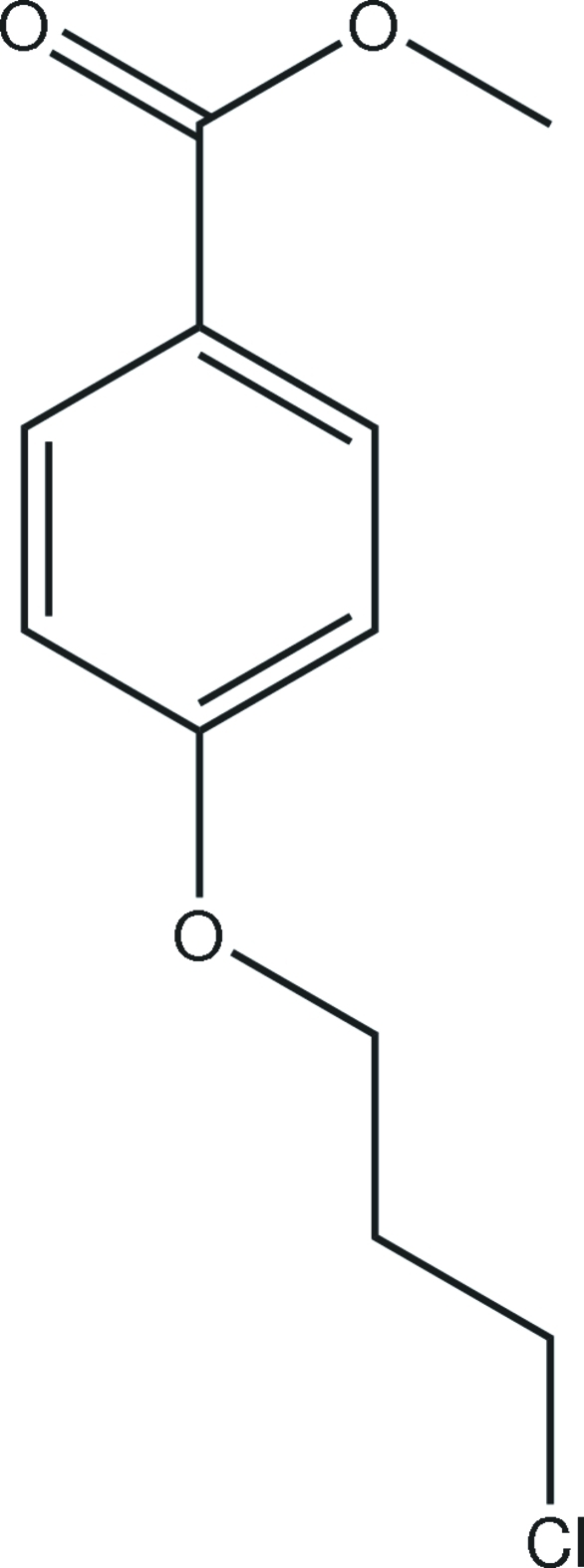


## Experimental

### 

#### Crystal data

C_11_H_13_ClO_3_
*M* *_r_* = 228.66
Monoclinic, 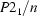

*a* = 6.2400 (12) Å
*b* = 10.611 (2) Å
*c* = 17.189 (3) Å
β = 100.35 (3)°
*V* = 1119.6 (4) Å^3^
*Z* = 4
Mo *K*α radiation
μ = 0.33 mm^−1^
*T* = 293 K
0.30 × 0.20 × 0.10 mm

#### Data collection

Enraf–Nonius CAD-4 diffractometer
Absorption correction: ψ scan (North *et al.*, 1968 ▶) *T*_min_ = 0.909, *T*_max_ = 0.968
4391 measured reflections
2063 independent reflections
1470 reflections with *I* > 2σ(*I*)
*R*_int_ = 0.045
3 standard reflections every 200 reflections  intensity decay: 1%

#### Refinement

*R*[*F* ^2^ > 2σ(*F* ^2^)] = 0.044
*wR*(*F* ^2^) = 0.132
*S* = 1.00
2063 reflections
137 parameters
H-atom parameters constrained
Δρ_max_ = 0.24 e Å^−3^
Δρ_min_ = −0.19 e Å^−3^

### 

Data collection: *CAD-4 EXPRESS* (Enraf–Nonius, 1989) ▶; cell refinement: *CAD-4 EXPRESS*; data reduction: *XCAD4* (Harms & Wocadlo, 1995 ▶); program(s) used to solve structure: *SHELXS97* (Sheldrick, 2008 ▶); program(s) used to refine structure: *SHELXL97* (Sheldrick, 2008 ▶); molecular graphics: *SHELXTL* (Sheldrick, 2008 ▶); software used to prepare material for publication: *PLATON* (Spek, 2009 ▶).

## Supplementary Material

Crystal structure: contains datablocks global, I. DOI: 10.1107/S1600536811011093/sj5116sup1.cif

Structure factors: contains datablocks I. DOI: 10.1107/S1600536811011093/sj5116Isup2.hkl

Additional supplementary materials:  crystallographic information; 3D view; checkCIF report

## Figures and Tables

**Table 1:** Hydrogen-bond geometry (Å, °)

*D*—H⋯*A*	*D*—H	H⋯*A*	*D*⋯*A*	*D*—H⋯*A*
C1—H1*A*⋯O2^i^	0.97	2.45	3.351 (3)	154

Symmetry code: (i) 
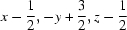
.

**Fig. 1.**
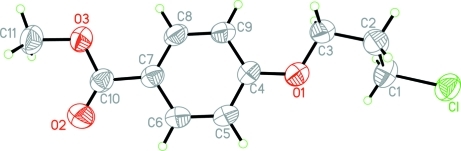
The molecular structure of the title molecule, with the atom-numbering scheme. Displacement ellipsoids are drawn at the 50% probability level.

**Fig. 2.**
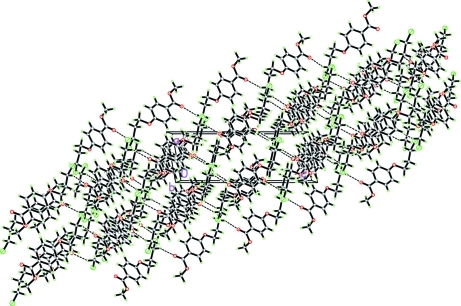
A packing diagram of (I) viewed down the b axis. Hydrogen bond are drawn as dashed lines.

## supplementary crystallographic information

### Comment

The title compound, methyl 4-(3-chloropropoxy)benzoate, is a useful pharmaceutical intermediate in the preparation of precursors to dronedarone. (Jaseer *et al.*, 2010).

We report here in the crystal structure of the title compound, methyl 2-amino-5-chlorobenzoate. In the molecule of the title compound (Fig. 1), the bond lengths (Allen *et al.*, 1987) and angles are within normal ranges. In the crystal structure, intermolecular C-H1A···O2 hydrogen bonds link the molecules into zig-zag chains along the *c* axis, to form a stable structure (Fig. 2).

### Experimental

The title compound, methyl 4-(3-chloropropoxy)benzoate was prepared by a literature method (Jaseer *et al.* 2010). Crystals suitable for X-ray analysis were obtained by slow evaporation of an ethyl acetate solution.

### Refinement

H atoms were positioned geometrically, with C-H = 0.93, 0.97 and 0.96 Å for aromatic, methylene and methyl H atoms, respectively, and constrained to ride on their parent atoms, with U_iso_(H) = xU_eq_(C,N), where x = 1.5 for methyl H and x = 1.2 for all other H atoms.

### Crystal data

**Table:**

C_11_H_13_ClO_3_	*F*(000) = 480
*M**_r_* = 228.66	*D*_x_ = 1.357 Mg m^−^^3^
Monoclinic, *P*2_1_/*n*	Melting point: 328 K
Hall symbol: -P 2yn	Mo *K*α radiation, λ = 0.71073 Å
*a* = 6.2400 (12) Å	Cell parameters from 25 reflections
*b* = 10.611 (2) Å	θ = 9–14°
*c* = 17.189 (3) Å	µ = 0.33 mm^−^^1^
β = 100.35 (3)°	*T* = 293 K
*V* = 1119.6 (4) Å^3^	Block, colourless
*Z* = 4	0.30 × 0.20 × 0.10 mm

### Data collection

**Table:**

Enraf–Nonius CAD-4 diffractometer	1470 reflections with *I* > 2σ(*I*)
Radiation source: fine-focus sealed tube	*R*_int_ = 0.045
graphite	θ_max_ = 25.4°, θ_min_ = 2.3°
ω/2θ scans	*h* = 0→7
Absorption correction: ψ scan (North *et al.*, 1968)	*k* = −12→12
*T*_min_ = 0.909, *T*_max_ = 0.968	*l* = −20→20
4391 measured reflections	3 standard reflections every 200 reflections
2063 independent reflections	intensity decay: 1%

### Refinement

**Table:**

Refinement on *F*^2^	Secondary atom site location: difference Fourier map
Least-squares matrix: full	Hydrogen site location: inferred from neighbouring sites
*R*[*F*^2^ > 2σ(*F*^2^)] = 0.044	H-atom parameters constrained
*wR*(*F*^2^) = 0.132	*w* = 1/[σ^2^(*F*_o_^2^) + (0.078*P*)^2^] where *P* = (*F*_o_^2^ + 2*F*_c_^2^)/3
*S* = 1.00	(Δ/σ)_max_ < 0.001
2063 reflections	Δρ_max_ = 0.24 e Å^−^^3^
137 parameters	Δρ_min_ = −0.19 e Å^−^^3^
0 restraints	Extinction correction: *SHELXL*, Fc^*^=kFc[1+0.001xFc^2^λ^3^/sin(2θ)]^-1/4^
Primary atom site location: structure-invariant direct methods	Extinction coefficient: 0.017 (3)

### Special details

**Table:**

Geometry. All esds (except the esd in the dihedral angle between two l.s. planes) are estimated using the full covariance matrix. The cell esds are taken into account individually in the estimation of esds in distances, angles and torsion angles; correlations between esds in cell parameters are only used when they are defined by crystal symmetry. An approximate (isotropic) treatment of cell esds is used for estimating esds involving l.s. planes.
Refinement. Refinement of F^2^ against ALL reflections. The weighted R-factor wR and goodness of fit S are based on F^2^, conventional R-factors R are based on F, with F set to zero for negative F^2^. The threshold expression of F^2^ > 2sigma(F^2^) is used only for calculating R-factors(gt) etc. and is not relevant to the choice of reflections for refinement. R-factors based on F^2^ are statistically about twice as large as those based on F, and R- factors based on ALL data will be even larger.

### Fractional atomic coordinates and isotropic or equivalent isotropic displacement parameters (Å^2^)

**Table:**

	*x*	*y*	*z*	*U*_iso_*/*U*_eq_	
Cl	0.18566 (11)	0.25211 (6)	0.23318 (4)	0.0652 (3)	
O1	0.6595 (3)	0.50541 (15)	0.39148 (9)	0.0564 (5)	
C1	0.3616 (4)	0.3834 (2)	0.26514 (16)	0.0607 (7)	
H1A	0.3635	0.4397	0.2208	0.073*	
H1B	0.3046	0.4298	0.3056	0.073*	
O2	1.0386 (3)	0.94288 (18)	0.62662 (11)	0.0759 (6)	
C2	0.5887 (4)	0.3415 (2)	0.29744 (15)	0.0580 (6)	
H2A	0.6463	0.2964	0.2566	0.070*	
H2B	0.5861	0.2838	0.3410	0.070*	
O3	1.3033 (3)	0.92169 (16)	0.55628 (10)	0.0597 (5)	
C3	0.7360 (4)	0.4500 (2)	0.32576 (14)	0.0560 (6)	
H3A	0.8847	0.4207	0.3418	0.067*	
H3B	0.7328	0.5115	0.2838	0.067*	
C4	0.7766 (3)	0.5999 (2)	0.43188 (13)	0.0455 (5)	
C5	0.6944 (4)	0.6495 (2)	0.49502 (14)	0.0536 (6)	
H5A	0.5652	0.6180	0.5071	0.064*	
C6	0.8022 (4)	0.7447 (2)	0.53971 (14)	0.0520 (6)	
H6A	0.7446	0.7779	0.5816	0.062*	
C7	0.9978 (3)	0.7924 (2)	0.52298 (12)	0.0444 (5)	
C8	1.0772 (4)	0.7424 (2)	0.45955 (13)	0.0494 (6)	
H8A	1.2066	0.7736	0.4475	0.059*	
C9	0.9695 (4)	0.6480 (2)	0.41406 (13)	0.0520 (6)	
H9A	1.0253	0.6162	0.3714	0.062*	
C10	1.1089 (4)	0.8931 (2)	0.57411 (13)	0.0488 (5)	
C11	1.4283 (4)	1.0149 (2)	0.60542 (15)	0.0660 (7)	
H11A	1.5645	1.0279	0.5881	0.099*	
H11B	1.4553	0.9864	0.6593	0.099*	
H11C	1.3485	1.0927	0.6017	0.099*	

### Atomic displacement parameters (Å^2^)

**Table:**

	*U*^11^	*U*^22^	*U*^33^	*U*^12^	*U*^13^	*U*^23^
Cl	0.0545 (4)	0.0718 (5)	0.0679 (4)	−0.0158 (3)	0.0069 (3)	−0.0037 (3)
O1	0.0519 (10)	0.0640 (11)	0.0561 (9)	−0.0138 (8)	0.0172 (8)	−0.0071 (8)
C1	0.0523 (14)	0.0559 (14)	0.0691 (16)	−0.0074 (12)	−0.0018 (12)	0.0098 (12)
O2	0.0740 (13)	0.0836 (13)	0.0768 (12)	−0.0177 (10)	0.0311 (11)	−0.0280 (10)
C2	0.0509 (14)	0.0593 (14)	0.0628 (14)	0.0006 (11)	0.0079 (12)	−0.0079 (11)
O3	0.0512 (10)	0.0704 (11)	0.0576 (10)	−0.0181 (8)	0.0101 (8)	−0.0101 (8)
C3	0.0453 (13)	0.0651 (15)	0.0586 (14)	−0.0031 (11)	0.0121 (11)	−0.0075 (11)
C4	0.0407 (12)	0.0467 (12)	0.0490 (12)	−0.0051 (10)	0.0080 (10)	0.0032 (10)
C5	0.0455 (13)	0.0605 (14)	0.0592 (13)	−0.0101 (11)	0.0214 (11)	−0.0004 (12)
C6	0.0490 (13)	0.0584 (14)	0.0524 (13)	−0.0049 (11)	0.0195 (11)	−0.0010 (11)
C7	0.0389 (11)	0.0507 (12)	0.0428 (11)	0.0003 (10)	0.0054 (9)	0.0071 (10)
C8	0.0380 (12)	0.0580 (13)	0.0538 (13)	−0.0081 (11)	0.0128 (10)	0.0027 (11)
C9	0.0447 (13)	0.0645 (14)	0.0501 (12)	−0.0056 (11)	0.0172 (10)	−0.0036 (11)
C10	0.0445 (13)	0.0535 (13)	0.0478 (12)	−0.0042 (11)	0.0068 (10)	0.0072 (10)
C11	0.0602 (16)	0.0682 (17)	0.0667 (16)	−0.0193 (14)	0.0035 (13)	−0.0076 (13)

### Geometric parameters (Å, °)

**Table:**

Cl—C1	1.798 (2)	C4—C5	1.385 (3)
O1—C4	1.356 (3)	C4—C9	1.391 (3)
O1—C3	1.430 (3)	C5—C6	1.370 (3)
C1—C2	1.494 (3)	C5—H5A	0.9300
C1—H1A	0.9700	C6—C7	1.398 (3)
C1—H1B	0.9700	C6—H6A	0.9300
O2—C10	1.195 (3)	C7—C8	1.382 (3)
C2—C3	1.499 (3)	C7—C10	1.476 (3)
C2—H2A	0.9700	C8—C9	1.370 (3)
C2—H2B	0.9700	C8—H8A	0.9300
O3—C10	1.338 (3)	C9—H9A	0.9300
O3—C11	1.436 (3)	C11—H11A	0.9600
C3—H3A	0.9700	C11—H11B	0.9600
C3—H3B	0.9700	C11—H11C	0.9600
			
C4—O1—C3	118.88 (17)	C6—C5—H5A	119.8
C2—C1—Cl	111.69 (17)	C4—C5—H5A	119.8
C2—C1—H1A	109.3	C5—C6—C7	120.7 (2)
Cl—C1—H1A	109.3	C5—C6—H6A	119.7
C2—C1—H1B	109.3	C7—C6—H6A	119.7
Cl—C1—H1B	109.3	C8—C7—C6	118.3 (2)
H1A—C1—H1B	107.9	C8—C7—C10	123.4 (2)
C1—C2—C3	112.2 (2)	C6—C7—C10	118.3 (2)
C1—C2—H2A	109.2	C9—C8—C7	121.5 (2)
C3—C2—H2A	109.2	C9—C8—H8A	119.3
C1—C2—H2B	109.2	C7—C8—H8A	119.3
C3—C2—H2B	109.2	C8—C9—C4	119.8 (2)
H2A—C2—H2B	107.9	C8—C9—H9A	120.1
C10—O3—C11	116.09 (19)	C4—C9—H9A	120.1
O1—C3—C2	107.46 (19)	O2—C10—O3	123.0 (2)
O1—C3—H3A	110.2	O2—C10—C7	125.0 (2)
C2—C3—H3A	110.2	O3—C10—C7	112.1 (2)
O1—C3—H3B	110.2	O3—C11—H11A	109.5
C2—C3—H3B	110.2	O3—C11—H11B	109.5
H3A—C3—H3B	108.5	H11A—C11—H11B	109.5
O1—C4—C5	116.15 (19)	O3—C11—H11C	109.5
O1—C4—C9	124.5 (2)	H11A—C11—H11C	109.5
C5—C4—C9	119.4 (2)	H11B—C11—H11C	109.5
C6—C5—C4	120.4 (2)		
			
Cl—C1—C2—C3	−178.88 (17)	C10—C7—C8—C9	179.4 (2)
C4—O1—C3—C2	174.81 (19)	C7—C8—C9—C4	−0.5 (3)
C1—C2—C3—O1	65.1 (3)	O1—C4—C9—C8	−178.9 (2)
C3—O1—C4—C5	−179.9 (2)	C5—C4—C9—C8	0.8 (3)
C3—O1—C4—C9	−0.1 (3)	C11—O3—C10—O2	1.4 (3)
O1—C4—C5—C6	179.5 (2)	C11—O3—C10—C7	−177.2 (2)
C9—C4—C5—C6	−0.2 (3)	C8—C7—C10—O2	175.7 (2)
C4—C5—C6—C7	−0.7 (4)	C6—C7—C10—O2	−4.4 (4)
C5—C6—C7—C8	1.0 (3)	C8—C7—C10—O3	−5.7 (3)
C5—C6—C7—C10	−178.8 (2)	C6—C7—C10—O3	174.1 (2)
C6—C7—C8—C9	−0.4 (3)		

### Hydrogen-bond geometry (Å, °)

**Table:**

*D*—H···*A*	*D*—H	H···*A*	*D*···*A*	*D*—H···*A*
C1—H1A···O2^i^	0.97	2.45	3.351 (3)	154

Symmetry codes: (i) *x*−1/2, −*y*+3/2, *z*−1/2.
